# Hepcidin as a Sensitive and Treatment-Responsive Acute-Phase Marker in Patients with Bacteremia: A Pilot Study

**DOI:** 10.3390/diagnostics12061404

**Published:** 2022-06-06

**Authors:** Kreon Koukoulas, Vasiliki Lygoura, Philip Kartalidis, Nikolaos K. Gatselis, Efthymia Petinaki, George N. Dalekos, George Simos

**Affiliations:** 1 Laboratory of Biochemistry, Faculty of Medicine, University of Thessaly, 41500 Larissa, Greece; kkoukoulas@uth.gr; 2 Department of Medicine & Research Laboratory of Internal Medicine, National Expertise Center of Greece in Autoimmune Liver Diseases, European Reference Network on Hepatological Diseases (ERN RARE-LIVER), General University Hospital of Larissa, 41100 Larissa, Greece; vlygoura@gmail.com (V.L.); gatselis@me.com (N.K.G.); 3 Department of Medical Biopathology, Faculty of Medicine, University of Thessaly, 41100 Larissa, Greece; kartalidisphilip@gmail.com (P.K.); petinaki@med.uth.gr (E.P.)

**Keywords:** hepcidin, bacteremia, ferritin, CRP, acute phase marker

## Abstract

Hepcidin regulates iron metabolism by inhibiting intestinal iron absorption and iron release from iron stores. In addition to iron overload, inflammatory conditions also up-regulate hepcidin synthesis, which may serve as an antimicrobial defense by reducing iron availability to the invading microbes. The purpose of this study is to test this hypothesis in human patients by determining serum hepcidin concentration by enzyme linked immunosorbent assay (ELISA) in healthy blood donors (*n* = 60) and patients hospitalized because of bacteremia (*n* = 50), before (day 0) and after seven days (day 7) of appropriate antibiotic treatment. Serum hepcidin was significantly increased in patients with bacteremia, both at day 0 and at day 7, compared to healthy controls. However, there was significant reduction of serum hepcidin after 7-day treatment, in concert with changes in serum C-reactive protein (CRP). The hepcidin changes were similar for both Gram-negative and Gram-positive single infection cases, while CRP was significantly reduced only in the former. In contrast to hepcidin, the levels of serum ferritin in the patients remained high after treatment, irrespective of infection type. These data confirm the stimulation of hepcidin secretion in human subjects upon different types of systemic microbial infection and suggest that hepcidin is a more sensitive and treatment-responsive acute-phase marker than ferritin in bacteremia, which needs to be explored with bigger-sized and better-matched patient cohorts.

## 1. Introduction

Hepcidin is a peptide hormone predominantly synthesized and secreted by hepatocytes [1]. Hepcidin binds to ferroportin, the main cellular iron export channel protein in mammalian cells, and promotes its internalization and subsequent degradation [2]. Hepcidin absence can lead to iron overload, as it was reported particularly in juvenile hereditary hemochromatosis [3]. On the other hand, high levels of hepcidin cause iron retention in iron storage cells such as hepatocytes, enterocytes, and macrophages and, subsequently, diminish duodenal absorption and release of iron in the bloodstream [4]. Accordingly, hepcidin expression is stimulated by iron loading and is suppressed by the stimulation of erythropoiesis due to anemia or hypoxia [5].

Inflammation and infections can also stimulate hepcidin synthesis by hepatocytes and Kupffer’s cells via the interleukin-6 (IL-6) pathway, leading to the reduction of iron plasma concentration. In this respect, animal studies have suggested that hepcidin contributes to host defense by depriving invading microbes access to iron [6]. However, there have been relatively few studies in human subjects investigating the role of serum hepcidin upon systemic infections or inflammation.

In an early study, serum hepcidin was increased in a cohort of septic patients, 20% of whom had bacteremia as attested by a positive blood culture [7], and more recently, serum hepcidin was reported as elevated in 27 patients with bacteremia, compared to the non-bacterial infection group of patients with systemic inflammatory response syndrome [8], and in 15 patients with septic shock [9]. Serum hepcidin was also increased in septic infants compared with infants without sepsis [10], while in a more recent study with febrile children, serum hepcidin was elevated in the small number of children with bacterial infection [11].

The purpose of this study is to address the involvement of serum hepcidin and examine its diagnostic value in systemic infections. To do this, we determined serum hepcidin in a cohort of 50 hospitalized patients with proven bacteremia, before and after seven days of appropriate antibiotic treatment, and we evaluated whether hepcidin alterations were associated with the type of the bacterial strain responsible for the systemic infection. We also compared and correlated hepcidin levels with the corresponding values of two routinely tested acute phase proteins, namely ferritin and CRP, and determined the hepcidin/logferritin ratio. Ferritin is also a marker of iron stores and correlates positively with serum hepcidin in the general population [12]. As iron load triggers both ferritin and hepcidin production, the hepcidin/logferritin ratio remains stable in healthy individuals but alters upon deregulation of the iron–hepcidin axis and upon inflammation or autoimmune disorders [13]. To determine the normal range of serum hepcidin and the hepcidin/logferritin ratio in our relevant healthy population, we measured serum hepcidin and ferritin in 60 healthy blood donors.

## 2. Materials and Methods

### 2.1. Patients and Samples

This study included 25 consecutive male and 25 consecutive female patients, who were admitted to the Department of Medicine and Research Laboratory of Internal Medicine, General University Hospital of Larissa, Larissa, Greece, because of suspected symptoms of serious underlying systemic infection; who were not previously treated with any antimicrobial agent; and who were proven to suffer from bacteremia, as attested by a positive blood culture. Sixty apparently healthy volunteer blood donors (30 males and 30 females) were used as healthy controls. The Scientific Committee of the General University Hospital of Larissa approved the research protocol (approval number: 18865/21.05.2019), confirming that the study was planned in accordance with the Declaration of Helsinki. All patients and healthy controls gave informed consent for participation in the study.

Blood samples were collected from the patient group upon the day of the blood culture (day 0) and after a seven-day period (day 7), during which they received adequate and appropriate, according to local guidelines, antibiotic treatment. These samples, as well as the blood samples from healthy controls, were immediately centrifuged, and sera were separated into aliquots and stored at −80 °C for future assays. Relevant demographic, clinical, biochemical, and microbiological data were determined as part of our standard hospital care and were retrieved from the patients’ records.

Serum levels of hepcidin were determined by ELISA. The ELISA kits used were commercially available sandwich enzyme immunoassays using synthetic hepcidin (Quantikine ELISA, Human Hepcidin Immunoassay, Catalog Number: DHP250, R&D Systems, Minneapolis, MN, USA).

### 2.2. Statistical Analysis

Statistical analyses were performed using GraphPad Prism 5.0 (GraphPad Software, Inc, San Diego, CA, USA). A Shapiro–Wilk test was conducted to assess the normality of the value distribution. The distribution was found to be non-normal in all measured parameters except age and serum ferritin in the healthy group; therefore, non-parametric tests were performed to evaluate the differences and correlation between the different parameters. The values of different parameters were compared using the Mann–Whitney test for unpaired groups (Healthy vs. Bacteremia) and the Wilcoxon signed-rank test for paired groups (Bacteremia day 0 vs. day 7). A linear regression analysis was conducted to evaluate the relation between serum hepcidin and other relevant parameters. Correlations were calculated using Spearman’s correlation test. Age is expressed as mean ± standard deviation (SD), while serum analyses values are expressed as the median (interquartile range; IQR). Two-sided *p*-values < 0.05 were considered as statistically significant.

## 3. Results

The relevant demographic and clinical characteristics of healthy controls and the patients are summarized in Table 1. The mean age of healthy controls was significantly lower compared to that of the patients (Table 1; *p* < 0.0001).

**Figure 1:**
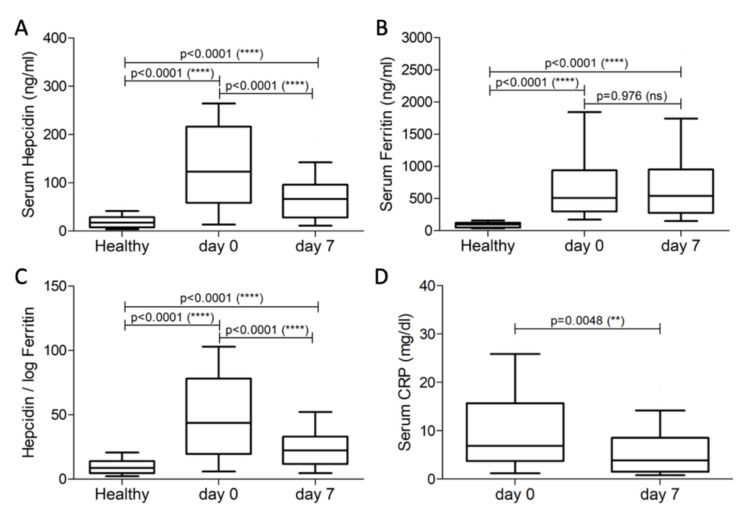
Comparison between healthy subjects and bacteremia patients on day 0 or 7. Serum Hepcidin (**A**), Serum Ferritin (**B**), Hepcidin /log Ferritin (**C**), and Serum CRP (**D**) in the studied groups. ns: non-significant; **: *p* < 0.005; ****: *p* < 0.0001.

Serum hepcidin in the patients was significantly higher than healthy controls, both on day 0 and day 7 (*p* < 0.0001 for both comparisons; Table 1 and Figure 1A). However, serum hepcidin in the bacteremia group was significantly reduced after the seven-day treatment (*p* < 0.0001; Table 1 and Figure 1A). Similar to serum hepcidin, serum ferritin levels in the patients were higher than the healthy controls, both on day 0 and day 7 (*p* < 0.0001; Table 1 and Figure 1B). However, unlike serum hepcidin, serum ferritin levels in patients were almost identical on day 0 and day 7: 509 (641) ng/mL vs. 542 (674) ng/mL (*p* = 0.976).

The ratio hepcidin/logferritin was significantly higher in the bacteremia patients compared to healthy controls, both on day 0 and day 7 *(p* < 0.0001; Table 1 and Figure 1C). Following the hepcidin trend, the ratio of hepcidin/logferritin was significantly reduced on day 7 compared to day 0 (*p* < 0.0001; Table 1 and Figure 1C). CRP serum levels were determined only in the bacteremia group of patients, and they were higher than the normal range (0.8–1.0 mg/dL or lower), both on day 0 and day 7, despite their significant reduction during treatment (*p* = 0.0048; Table 1 and Figure 1D).

The microbes isolated from the bacteremia patients are shown in Table 2. As determined by the blood culture results, 44 (88%) of the patients suffered from systemic infection by a single microbe, while in the remaining 6 (12%) patients, two different bacterial strains were isolated and identified.

Most of the single infections (31/50; 62%) were due to Gram-negative bacteria, while Gram-positive bacteria were identified in 13/50 (26%) of the patients. The commonest strains were *E. coli* (in 11 single-infections and 2 patients with co-infections) and *Enterococcus* species (in 8 single-infections and 2 patients with co-infections). Among the single bacterial infection subgroups, the size of which allowed statistical analysis, i.e., *E. coli* (*n* = 11), Gram-negative strains (*n* = 31), *Enterococcus* species (*n* = 7), and Gram-positive strains (*n* = 13), serum hepcidin was elevated on day 0 compared to those of the healthy controls (*p* = 0.0001–0.0002) and was significantly reduced on day 7 (*p* = 0.001, 0.0004, 0.0156, and 0.0007, respectively) (Table 3).

In the few cases of infections caused by other strains, there was either no initial increase in serum hepcidin (e.g., by *E. cloacae*) or no subsequent decrease (e.g., *Klebsiella* and *Acinetobacter* species) (Table 2). However, the small number of patients infected by these strains does not allow for any reliable conclusions.

As in the whole patient group, there were no significant changes of serum ferritin between day 0 and day 7 in the aforementioned single infection groups (Table 3). On the other hand, changes in serum CRP levels followed those of serum hepcidin for the Gram-negative bacterial groups, although with weaker statistical significance, while in the Gram-positive bacterial groups, there was no significant change of CRP after treatment (Table 3). Interestingly, in the small group of co-infections (*n* = 6), changes in all three markers (hepcidin, ferritin, and CRP) after treatment were statistically insignificant.

Correlations between the measured variables in the same subjects are shown in Table 4. In the healthy controls, there was a weak positive correlation between serum hepcidin and age, which, however, was not observed in the patient group. As expected, there was a strong positive correlation between serum hepcidin and ferritin levels in the healthy controls. This correlation was largely lost in the bacteremia patients before treatment but became again significant in the same patients after treatment. In contrast, there was a weak but significant positive correlation between serum hepcidin and CRP levels in the patient group both before and after treatment.

## 4. Discussion

Hepcidin synthesis by the hepatocytes is controlled by the Bone morphogenetic protein (BMP)—Small Mothers Against Decapentaplegic (SMAD)1/5/8 signaling pathway in response to body iron levels. Disorders in this pathway can lead to iron overload or hypoferremia. In addition, hepcidin synthesis is activated upon inflammation by the IL6—Janus Kinase (JAK)2—Signal Transducer and Activator of Transcription (STAT)3 signaling pathway, being, therefore, considered a type II acute phase protein [1].

Hepcidin was originally identified as a novel antimicrobial peptide displaying a β-defensin-like structure [14], but soon after its discovery, it was shown that it is a major regulator of iron homeostasis [15,16]. Its upregulation by inflammation is thought to serve as an innate defense mechanism against systemic bacterial infections, exploiting both the modest antimicrobial properties of hepcidin and its negative effect on circulating iron levels. The latter, as is the case of ferritin, another acute phase protein, can limit the availability of iron to the invading pathogens and curtail their proliferation. Indeed, studies in animals have shown that hepcidin contributes to the host defense by depriving microbes of access to iron [6].

Although studies in human subjects are in favor of this scenario, as an increase in serum hepcidin has been reported in cases of septic patients or patients under chronic inflammatory conditions [7,9,17,18], a direct link between invading pathogens and hepcidin upregulation has not been easily or firmly established due to a lack of prospective studies. Retrospective studies cannot easily exclude the possibility that the high levels of hepcidin observed in patients are not a result of the disease, but rather of a preexisting condition caused by a disorder in iron homeostasis and related to the pathogenicity of or the susceptibility to the disease. We tried to take these issues into account when designing our study.

More specifically, in contrast to previous studies examining septic patients of different etiologies, our study included only patients with proven bacteremia, as shown by a positive blood culture and isolation of the bacterial pathogen, the identity of which was correlated to the levels of serum hepcidin. In addition, the same patients were analyzed after seven-day treatment with the appropriate antibiotic, which could only have reduced the bacterial load. We reasoned that changes in serum hepcidin during this short period could not have occurred because of changes in iron load and must be predominantly affected by changes in the bacterial load and the ensuing inflammatory status.

To summarize our results, patients with bacteremia caused by a single agent infection exhibited a many-fold increase in serum hepcidin, which was significantly subsided after antibiotic treatment, suggesting that hepcidin might be valuable for monitoring treatment success in patients with bacteremia. Both the initial increase and subsequent reduction occurred irrespective of the type of invading microbe, at least in the numerous groups, such as Gram-negative bacteria, with predominant *E. coli* frequency, and Gram-positive bacteria, with predominant *Enterococcus* species frequency. At least for these single-infection subgroups, the kinetics of serum hepcidin appeared to be similar, characterized by an initial increase that subsides after treatment, and there were also no discernible differences in serum hepcidin between the Gram-negative and Gram-positive groups. Interestingly, in the small group of co-infections, changes in all three markers (hepcidin, ferritin, and CRP) after treatment were statistically insignificant, perhaps as a result of persistent infection severity. To our knowledge, the current study is the first to assess the connection of serum hepcidin to the types of bacteria causing a systemic infection.

In agreement with previous recent studies [8,9,11], the changes in bacteremia patient serum hepcidin correlated well with the changes in CRP, except in the case of the fewer in number infections with Gram-positive bacteria, in which CRP was not significantly reduced after treatment. In contrast, the correlation of hepcidin to ferritin, which was significant in the healthy controls, was lost in the non-treated bacteremia patients. This, together with the significant increase in the hepcidin/logferritin ratio, indicates a disproportionate increase of hepcidin synthesis compared to the increase in ferritin caused by the systemic infection and the ensuing inflammation or even any underlying iron overload. Accordingly, the re-establishment of a “healthier” correlation between hepcidin and ferritin in the treated patients signifies a reduction in inflammation, in agreement with the reduction of CRP levels.

Overall, our results confirm the inflammatory stimulation of hepcidin synthesis in human subjects and support the hypothesis that hepcidin also contributes to host anti-microbial defense. Furthermore, hepcidin appears to be a more sensitive and treatment-responsive acute-phase marker than ferritin in patients with bacteremia and parallels or even exceeds the diagnostic accuracy of CRP. In fact, our findings with adult patients are in accordance with a recent study suggesting that hepcidin may be useful as a biomarker for identifying bacterial infections in febrile children [11]. Furthermore, a study with a small number of septic patients admitted to the intensive care unit reported a steady decline of serum hepcidin concentrations in these patients during their treatment, suggesting that hepcidin may also be a useful marker for assessing the efficacy of treatment in critically ill patients [9].

There are several unavoidable limitations to our study. This was a single-center study, though prospective, with a relatively small sample size, so the data need to be validated in a larger cohort of patients. The healthy and the patient group were not age-matched, as blood donors are usually much younger than patients with systemic infections. However, despite the weak positive correlation between serum hepcidin and age in our blood donor group, there was no such correlation in our patient group or in the other healthy groups analyzed in previous studies [19]. Therefore, it is highly unlikely that the high serum hepcidin levels in our bacteremia group prior to treatment were due to the older age of the patients. This is further supported by the decline of hepcidin after the administration of specific antibiotic treatment. There is no control patient group without treatment, as it is unethical to leave patients with bacteremia untreated. However, further studies need to use a control group of age-matched patients with non-infectious diseases, as this would contribute to a better understanding of the potential correlation of hepcidin with other variables and minimize the influence of confounding factors and co-morbidities connected with older age. Finally, another limitation of this study is that other iron-related biomarkers, such as free iron, transferrin, and transferrin saturation, were not analyzed, since the focus of our study was rather on infection and acute phase biomarkers than anemia or other iron metabolism disorders.

There is certainly need for additional studies that may establish serum hepcidin as a biomarker with superior diagnostic significance compared to the ones currently and routinely used in bacteremia or sepsis cases. This, as well as the inclusion of hepcidin measurements in the everyday routine clinical practice, will be undoubtfully facilitated by the development of automated and bedside serum hepcidin tests, which are still not widely available.

## 5. Conclusions

The present single-center, prospective, observational pilot study demonstrated the robust increase of serum hepcidin in patients with bacteremia caused by different microbial agents and its decline in response to adequate antibiotic treatment, mostly in correlation with CRP but not ferritin. Although clinical severity was not assessed in our study, it is possible that the determination of hepcidin may add valuable information to routinely used biomarkers for evaluating bacterial infections at baseline and during their course, after the administration of the appropriate antibiotic treatment. This needs to be addressed by further studies that will also correlate serum hepcidin to the severity of bacterial infections.

## Figures and Tables

**Table 1:** Demographic and clinical characteristics of healthy controls and patients with bacteremia on day 0 and day 7 after appropriate treatment. Values are expressed as the mean ± SD or median (IQR).

Variables	Healthy *n* = 60	Bacteremia *n* = 50, Day 0	Bacteremia *n* = 50, Day 7	*p*-Value
Age (years)	51 ± 6	74 ± 15	74 ± 15	<0.0001 ^a,b^
Sex, male/female	30/30	25/25	25/25	NA
Serum Hepcidin (ng/mL)	18 (21)	123 (158)	67 (68)	<0.0001 ^a,b,c^
Serum Ferritin (ng/mL)	95 (77)	509 (641)	542 (674)	<0.0001 ^a,b^ 0.9760 ^c^
Serum CRP (mg/dL)	NA	6.9 (11.9)	3.9 (7)	0.0048 ^c^
Serum Hepcidin/log ferritin	8.7 (9.4)	43.7 (58.7)	22.3 (21.2)	<0.0001 ^a,b,c^

NA, not applicable. ^a^ *p*-value refers to difference between healthy controls and bacteremia patients on day 0. ^b^ *p*-value refers to the difference between healthy controls and bacteremia patients on day 7. ^c^ *p*-value refers to difference between bacteremia patients on day 0 and day 7.

**Table 2:** Blood culture results. Values represent the median (IQR) when number of cases (n) > 3 and mean (range) when n ≤ 3.

Organism	Gram	n	Serum Hepcidin (ng/mL)	
			Day 0	Day 7
*Escherichia coli*	-ve	11	180 (103)	60 (66)
*Pseudomonas aeruginosa*	-ve	4	142 (272)	51 (85)
*Klebsiella*	-ve	3	81 (90)	90 (59)
*Brucella*	-ve	3	67 (119)	60 (74)
*Acinetobacter species*	-ve	3	62 (67)	88 (169)
*Citrobacter species*	-ve	2	146 (3)	62 (11)
*Proteus mirabilis*	-ve	1	360	263
*Enterobacter cloacae*	-ve	1	9	24
*Providencia stuartii*	-ve	1	227	77
*Morganella morganii*	-ve	1	343	180
*Salmonella*	-ve	1	260	137
Gram-negative TOTAL		31	133 (167)	75 (67)
*Enterococcus species*	+ve	7	142 (127)	61 (94)
*Streptococcus species*	+ve	2	96 (49)	37 (38)
*Staphylococcus aureus MS*	+ve	2	91 (102)	45 (4)
*Staphylococcus aureus MR*	+ve	2	26 (45)	20 (35)
Gram-positive TOTAL		13	120 (127)	47 (55)
Single infection TOTAL		44	128 (171)	61 (71)
*E. coli*/*Klebsiella*	-ve/-ve	1	234	99
*E. coli*/*P. mirabilis*	-ve/-ve	1	124	84
*A. baumannii*/*P. stuartii*	-ve/-ve	1	62	75
*A. baumannii*/*S. aureus*	-ve/+ve	1	77	45
*E. faecalis*/*S. aureus*	+ve/+ve	1	195	81
*E. faecalis*/*E. faecium*	+ve/+ve	1	48	134
Co-infection TOTAL		6	101 (146)	83 (40)
ALL INFECTIONS TOTAL		50	123 (158)	67 (68)

**Table 3:** Changes of measured variables between day 0 and day 7 in the subgroups of patients with different types of infection. Values are expressed as the median (IQR).

Organism	n	Hepcidin (ng/mL)		Ferritin (ng/mL)		CRP (mg/dL)	
		Day 0	Day 7	Day 0	Day 7	Day 0	Day 7
*Escherichia coli*	11	180 (103)	60 (66)	377 (503)	409 (691)	19.0 (19.1)	2.92 (2.8)
		*p* = 0.001		*p* = 0.3652		*p* = 0.0049	
Gram-negative	31	136 (169)	68 (67)	576 (749)	590 (909)	7.9 (16.2)	2.9 (8.5)
		*p* = 0.0004		*p* = 0.9925		*p* = 0.036	
*Enterococcus* species	7	142 (127)	61 (94)	811 (1482)	616 (1350)	6.3 (12.9)	7 (7.8)
		*p* = 0.0156		*p* = 0.1563		*p* =0.4688	
Gram-positive	13	120 (127)	47 (55)	418 (1067)	410 (418)	6.5 (7.7)	5.5 (4.4)
		*p* = 0.0007		*p* = 0.5186		*p* = 0.064	
Single infections	44	128 (171)	61 (71)	420 (706)	533 (835)	7.1 (12.9)	3.5 (7.3)
		*p* < 0.0001		*p* = 0.6036		*p* = 0.0068	
Co-infections	6	101 (146)	83 (40)	688 (414)	595 (435)	6.5 (6.7)	7.5 (6.1)
		*p* = 0.3125		*p* = 0.1875		*p* = 0.4375	
ALL INFECTIONS	50	123 (158)	67 (68)	509 (641)	542 (674)	6.9 (11.9)	3.9 (7)
		*p* < 0.0001		*p* = 0.976		*p* = 0.0048	

**Table 4:** Correlation of hepcidin levels with other variables in healthy controls and bacteremia patients on day 0 and day 7.

Variable	Healthy		Bacteremia Day 0		Bacteremia Day 7	
	*r*	*p*-Value	*r*	*p*-Value	*r*	*p*-Value
Age	0.29	0.028	−0.08	0.564	−0.10	0.507
Ferritin	0.63	<0.0001	0.28	0.052	0.65	<0.0001
CRP			0.32	0.027	0.4	0.005
